# Profiling bacterial community in upper respiratory tracts

**DOI:** 10.1186/s12879-014-0583-3

**Published:** 2014-11-13

**Authors:** Hana Yi, Dongeun Yong, Kyungwon Lee, Yong-Joon Cho, Jongsik Chun

**Affiliations:** School of Biosystem and Biomedial Science, Korea University, Seoul, Republic of Korea; Department of Public Health Science, Graduate School, Korea University, Seoul, Republic of Korea; Korea University Guro Hospital, Korea University, Seoul, Republic of Korea; Department of Laboratory Medicine and Research Institute of Bacterial Resistance, Yonsei University College of Medicine, Seoul, Republic of Korea; ChunLab, Inc., Seoul, Republic of Korea; School of Biological Sciences & Institute of Bioinformatics (BIOMAX), Seoul National University, Seoul, Republic of Korea

**Keywords:** Microbiome, Respiratory tract, Moraxella, Influenza, Oropharynx, Healthcare staff

## Abstract

**Background:**

Infection by pathogenic viruses results in rapid epithelial damage and significantly impacts on the condition of the upper respiratory tract, thus the effects of viral infection may induce changes in microbiota. Thus, we aimed to define the healthy microbiota and the viral pathogen-affected microbiota in the upper respiratory tract. In addition, any association between the type of viral agent and the resultant microbiota profile was assessed.

**Methods:**

We analyzed the upper respiratory tract bacterial content of 57 healthy asymptomatic people (17 health-care workers and 40 community people) and 59 patients acutely infected with influenza, parainfluenza, rhino, respiratory syncytial, corona, adeno, or metapneumo viruses using culture-independent pyrosequencing.

**Results:**

The healthy subjects harbored primarily *Streptococcus*, whereas the patients showed an enrichment of *Haemophilus* or *Moraxella*. Quantifying the similarities between bacterial populations by using Fast UniFrac analysis indicated that bacterial profiles were apparently divisible into 6 oropharyngeal types in the tested subjects. The oropharyngeal types were not associated with the type of viruses, but were rather linked to the age of the subjects. *Moraxella nonliquefaciens* exhibited unprecedentedly high abundance in young subjects aged <6 years. The genome of *M. nonliquefaciens* was found to encode various proteins that may play roles in pathogenesis.

**Conclusions:**

This study identified 6 oropharyngeal microbiome types. No virus-specific bacterial profile was discovered, but comparative analysis of healthy adults and patients identified a bacterium specific to young patients, *M. nonliquefaciens.*

**Electronic supplementary material:**

The online version of this article (doi:10.1186/s12879-014-0583-3) contains supplementary material, which is available to authorized users.

## Background

Recent culture-independent community analysis performed on the human microbiome has provided an overall picture of commensal microbial communities. Studies have revealed that diverse microbes occupying body habitats with strong niche specialization both within and among individuals [1]. In the case of the respiratory tract microbiome, a catalogue was initially established in 2009 [2] and then respiratory microorganisms were extensively characterized [3]-[8]. Collectively, studies to date have revealed that the respiratory tract harbors a homogenous microbiota that decreases in biomass from the upper to the lower tract [5], and that the lung microbiome resembles the oral microbiome, although these microbiomes are distinguished by the overrepresentation of distinct bacterial species in the lung [7]. As with other human-body habitats, the core microbiome of nasopharynx remains undefined because it varies substantially from person to person [3]. However, existence of core microbiome was observed despite the significant inter-individual variation [8]. One study reported that the microbial composition of the upper respiratory tract is typically unique to each person and it changes little over time [4].

Although the available evidence is not sufficiently strong, microbiome types are speculated to eventually affect a person’s risk of disease or response to distinct drugs [9]. The human microbiota is considered to benefit the host by promoting the differentiation of the mucosal structure and function, stimulating both the innate and adaptive immune systems, and providing “colonization resistance” against pathogen invasion [10]. Recently, the composition of the airway microbiota has been suggested to play roles in determining the presence and severity of diseases [11],[12]. For example, the clinical outcomes of respiratory infections caused by *Pseudomonas aeruginosa* vary depending on the diversity of the airway microbiota [13],[14], and a temporal loss of the diversity is linked to the development of ventilator-associated pneumonia and patient mortality [12],[13]. The importance of intact commensal microbiota was also demonstrated in viral infection, with the commensal microbiota composition critically regulating host immune response following influenza virus infection [15]. To reveal the links that exist between microbiome types and clinical traits, we have to first understand the diversity of the microbial community in target body sites.

Most respiratory tract infections are caused by viruses including rhinovirus, respiratory syncytial virus, parainfluenza virus, adenovirus, coronavirus, human metapneumovirus, and influenza virus. Infection by pathogenic viruses significantly changes the condition of the respiratory tract as a result of the epithelial damage caused by viral invasion itself and/or by inflammatory mediators produced by the host immune response [16]. Given, the pathophysiology and mechanism of local immune responses are virus specific [16], a virus-specific bacterial profile in the respiratory tract could potentially be characterized. Discovering any specific bacterial species that exhibits a tendency of opportunistic infection or co-infection in a viral species-dependent would benefit future preventive measures and current treatments. To date, no study has evaluated whether the composition of the respiratory microbiota changes in relation to the type of infectious virus.

In this study, our aim was to determine whether a viral infection-related bacterial profile exists in the respiratory tract and evaluate any disparities in the microbiota structure that develops depending on the infectious virus species. We used culture-independent high-throughput sequencing to analyze the bacterial content in the upper respiratory tract of patients and healthy asymptomatic people. We also examined the presence or absence of dissimilarities in the microbiota of hospital staff and community people.

## Methods

### Ethics statement

This study was approved by the Institutional Review Board of the Severance Hospital, Yonsei University Health System, Seoul, Korea (protocols 4-2010-0652, 4-2011-0159, and 4-2011-0862). Patients and healthy adults provided written informed consent to be enrolled. De-identified demographic data and clinical measures were taken from electronic medical record system. Additional file 1: Table S1 presents the list and features of samples used in this study.

### Subjects and sample collection

We selected 59 patients with confirmed acute viral infections from Yonsei University Hospital during a 30-month period (December 2010 to May 2013). The viral agents of the infections were confirmed using PCR by Yonsei University Hospital. The viruses included influenza (IF, n = 7), parainfluenza (PI, n = 24), rhino (RH, n = 8), respiratory syncytial (RS, n = 14), corona (CR, n = 4), adeno (AD, n = 1), and metapneumo (MP, n = 1) viruses. The upper respiratory tract samples were collected from patients’ oropharynx by using swabs and suspended in 1 mL of viral transport medium (VTM; Becton Dickinson Universal Viral Transport, USA). Sputum or nasopharyngeal aspirate was collected when available instead of swabs. Sputum samples were diluted with an equal volume of suspension medium and homogenized as described [17]. The upper respiratory tract samples were also obtained from healthy adults including 17 health-care workers (9 non-ICU and 8 ICU staff) and 40 community people. The 17 hospital staffs and 7 community people were recruited over the same period in Yonsei University Hospital (June 2011) and 33 community people were additionally recruited in the same hospital (June 2013). The oropharyngeal swabs were obtained using aseptic technique, suspended in VTM and transported to the laboratory for further processing. The samples were stored at −80°C until DNA extraction.

### DNA extraction, PCR, and pyrosequencing

DNA was extracted from 200 μL of samples by using a commercial microbial DNA isolation kit (Qiagen). The extracted DNA was amplified using primers targeting the V1 to V3 regions of the prokaryotic 16S rRNA gene by using methods described elsewhere [18]. DNA was sequenced by Chunlab Inc. (Seoul, Korea) by using a Roche/454 GS Junior system according to the manufacturer’s instructions. The processing of pyrosequencing data of 16S rRNA gene sequences were performed as described elsewhere [18]. Chimeric sequences were detected using UCHIME [19] and EzTaxon-e database (http://eztaxon-e.ezbiocloud.net; [20]) was used to taxonomically assign each pyrosequencing read.

### Phylogenetic analyses

Phylogenetic analyses of 16S rRNA gene sequences were performed using the neighbor-joining [21] tree method implemented in MEGA program [22]. An evolutionary distance matrix was generated for the neighbor-joining tree according to the model of Jukes and Cantor [23] and the resultant tree topologies were evaluated using bootstrap analyses [24].

### Genome analyses

The draft genome sequence of *Moraxella nonliquefaciens* DSM 6327^T^ was determined through paired-end shotgun sequencing performed by using the MiSeq system (Illumina) with 300× coverage. The sequencing reads were assembled using CLC genomics wb5 (CLCbio). Annotation, comparative genomic analyses and average nucleotide identity (ANI) calculation were performed as described [25].

### Statistical analyses

Random subsampling was conducted to normalize the data size to 3,000 reads, because the total number of reads that remained after pre-processing varied depending on the samples. All statistical analyses were performed using this subset. The Simpson diversity index [26] was calculated using the rRNA Database Project’s pyrosequencing pipeline (http://pyro.cme.msu.edu/). The overall phylogenetic distance between each pair of communities was estimated using the Fast UniFrac web interface (http://unifrac.colorado.edu/) [27] and visualized using principal coordinate analysis (PCoA) implemented in R program (http://www.r-project.org/). To compare microbiome structures based on categorical metadata, samples were pooled into binds (healthy/patient, male/female, VTM/aspirate/sputum, smoking/non-smoking, ages, causal viruses, etc.) and statistical significance tests were performed using R program. The significance of differences in bacterial profiles according to categorical metadata was determined using Hotelling’s *t* test. Significant bacterial taxa based on categorical metadata were identified using *q*-values after multiple testing correction [28] to eliminate false discovery rates. The difference in Shannon diversity index among categorical metadata was evaluated using Wilcox two-sample *t* test.

### Availability of supporting data

The 454 sequencing data supporting this article are available in the GenBank repository, SUB435282. The genome data of *M. nonliquefacience* is under submission to the DDBJ/EMBL/GenBank databases under accession No. PRJNA232737.

## Results

### Sequencing statistics

We sequenced 57 upper respiratory tract samples from healthy adults including 17 health-care workers (9 non-ICU and 8 ICU staff) and 40 community people. The 59 patients with confirmed acute viral infections with influenza (IF, n = 7), parainfluenza (PI, n = 24), rhino (RH, n = 8), respiratory syncytial (RS, n = 14), corona (CR, n = 4), adeno (AD, n = 1), and metapneumo (MP, n = 1) viruses were also successfully sequenced. The pyrosequencing of 16S rRNA gene amplicons resulted in 786,152 quality-filtered reads for the 116 samples. We observed an average of 823 bacterial phylotypes (97% clustering) for each samples (range 237 to 1,851). In the sample-size-normalized (3,000 nt) subsamples, the number of bacterial species ranged from 180 to 615 (average, 371), depending on samples.

### Microbiota of healthy adults

The genus *Streptococcus* was identified as the core microbiome of the healthy human respiratory tract. In all healthy subjects tested in this study, members of *Streptococcus* dominated the bacterial community, exhibiting an average abundance ratio (percentage of the taxon in the total bacterial community) of 55.8% (range 13.4%-91.1%, depending on subjects) (Table 1). The genera *Neisseria* (8.0%) and *Gemella* (5.3%) were also dominant in healthy subjects, but their abundance ratios were considerably less than that of *Streptococcus.* The genera observed in all healthy subjects were *Streptococcus, Prevotella*, and *Veillonella*. The genera *Haemophilus*, *Gemella*, *Rothia*, and *Leptotrichia* were detected in most subjects at abundance ratios of 2.0%-8.0%.

**Table 1:** **List of dominant bacterial genera and their average abundance (%) in samples**

	Healthy adults				Patients							
	non ICU	ICU staff	Community	Total	IF	PI	RH	RS	CR	AD	MP	Total
	(n =9)	(n =8)	(n =40)	(n =57)	(n =7)	(n =24)	(n =8)	(n =14)	(n =4)	(n =1)	(n =1)	(n =59)
*Streptococcus*	32.1 ± 19.6	57.4 ± 19.8	60.8 ± 21.4	55.8	43.4 ± 28.3	23.3 ± 34.3	52.5 ± 20.3	35.1 ± 32.2	26.3 ± 32.6	0.6	43.8	32.6
*Haemophilus*	6.4 ± 4.9	6.1 ± 5.1	6.3 ± 7.9	6.3	11.5 ± 26.4	39.6 ± 43.1	7.2 ± 11.8	22.3 ± 37.2	0.1 ± 0.2	84.7	0.5	25.2
*Neisseria*	13.6 ± 13.4	8.1 ± 6.8	6.7 ± 9.6	8.0	8.2 ± 12.4	0.5 ± 1.1	2.5 ± 4.6	0.7 ± 2.0	0.1 ± 0.1	0.0	1.6	1.7
*Moraxella*	0.0 ± 0.0	0.0 ± 0.0	0.0 ± 0.0	0.0	0.3 ± 0.7	9.4 ± 23.2	9.3 ± 26.3	21.6 ± 35.0	6.6 ± 13.2	14.1	0.0	10.9
*Prevotella*	8.5 ± 7.4	0.6 ± 0.4	4.0 ± 7.1	4.2	5.6 ± 6.3	1.8 ± 6.1	8.0 ± 9.3	1.3 ± 2.8	0.7 ± 0.8	0.1	9.5	3.0
*Veillonella*	4.7 ± 3.7	1.2 ± 0.7	2.3 ± 3.3	2.5	8.6 ± 9.1	1.5 ± 4.6	4.2 ± 5.6	2.1 ± 3.6	7.3 ± 12.0	0.1	11.2	3.4
*Gemella*	5.6 ± 8.6	11.4 ± 9.5	4.0 ± 7.4	5.3	0.3 ± 0.6	0.3 ± 1.2	1.5 ± 2.9	0.1 ± 0.3	0.2 ± 0.4	0.0	0.4	0.4
*Klebsiella*	0.0 ± 0.0	0.0 ± 0.0	0.0 ± 0.0	0.0	0.0 ± 0.0	6.5 ± 22.1	0.0 ± 0.1	4.2 ± 8.6	0.0 ± 0.0	0.0	0.0	3.7
*Granulicatella*	1.3 ± 1.2	2.7 ± 2.4	0.8 ± 0.9	1.1	4.6 ± 4.7	0.6 ± 2.1	0.8 ± 1.0	3.9 ± 11.4	0.0 ± 0.1	0.0	5.4	1.9
*Rothia*	0.1 ± 0.1	1.4 ± 1.5	2.7 ± 3.7	2.1	0.3 ± 0.4	0.2 ± 0.7	3.3 ± 5.4	0.3 ± 0.9	0.0 ± 0.1	0.0	1.1	0.7
*Leptotrichia*	4.8 ± 9.7	0.3 ± 0.3	1.7 ± 5.5	2.0	0.8 ± 0.8	0.2 ± 0.9	0.1 ± 0.2	0.1 ± 0.3	0.4 ± 0.5	0.0	7.3	0.4
*Porphyromonas*	1.7 ± 2.2	2.9 ± 5.0	1.4 ± 2.8	1.7	2.8 ± 3.4	0.0 ± 0.1	0.2 ± 0.2	0.1 ± 0.3	0.0 ± 0.0	0.0	0.8	0.4
*Staphylococcus*	0.1 ± 0.2	0.0 ± 0.0	0.0 ± 0.0	0.0	0.1 ± 0.1	1.0 ± 3.6	2.5 ± 7.0	0.4 ± 1.1	17.1 ± 34.2	0.0	0.0	2.0

*Abbreviations*: *ICE* intensive care unit, *non ICU* healthy hospital staff who do not work in ICU, *ICU staff* healthy hospital staff who work in ICU, *Community* healthy community people, *IF* influenza, *PI* parainfluenza, *RH* rhino, *RS* respiratory syncytial, *CR* corona, *AD* adeno *MP* metapneumo.

Fast UniFrac analyses for the bacterial profiles in healthy samples showed that hospital staff and community people were not discriminated based on their bacterial composition (Additional file 2: Figure S1), and age, sex, year and month of sample collection, and smoking status did not discriminate the bacterial profile (data not shown).

### Difference between patient and healthy-adult groups

**Figure 1:**
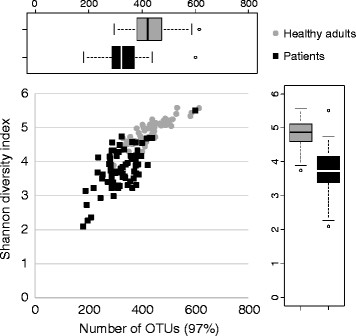
**Bacterial diversity observed in healthy-adult and patient groups represented by Shannon index and number of phylotypes (97% clustering).** Healthy people contained more diverse bacterial communities in their upper respiratory tract than did patients.

Analyzing the bacterial communities of healthy-adult and patient groups revealed clear differences. We used the Shannon index in which higher values represent higher diversities; the average values calculated for the healthy-adult and patient groups were 4.83 ± 0.40 and 3.77 ± 0.61, respectively (Figure 1). This indicated that healthy adults harbored significantly more complex and diverse microbiota than did patients (*p* <0.0001, Wilcox test).

**Figure 2:**
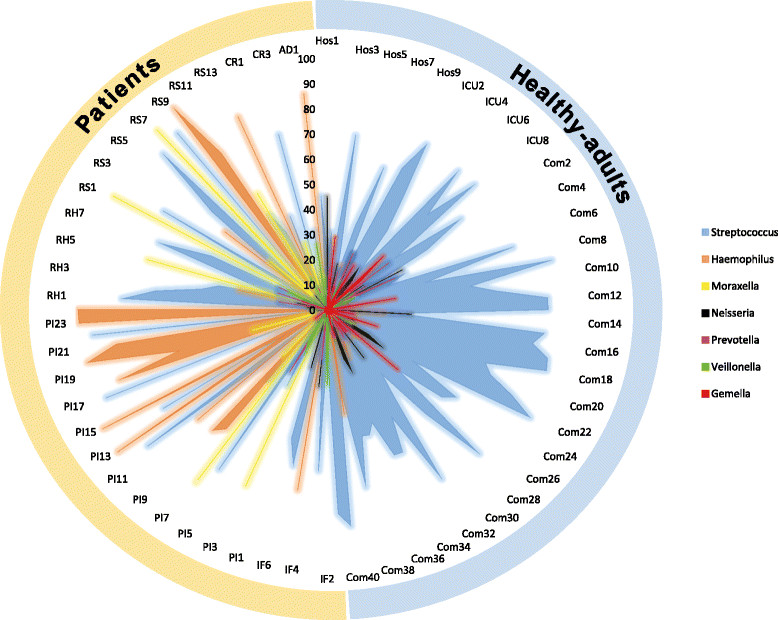
**Distinct populations of multiple genera observed in healthy-adult and patient groups.** The 7-most dominant genera observed in the samples were selected and are depicted in the radiation diagram. The height of each peak represents the percent ratio of the corresponding genus in a sample. *Streptococcus* and *Gemella* dominated in the healthy-adult group, whereas the genera *Haemophilus* and *Moraxella* dominated in the patient group.

The microbiota profiles of healthy-adult and patient groups also differed in the relative composition of the microbiome, which was highlighted in the graph showing the abundant bacterial genera observed in the tested samples (Figure 2 and Additional file 3: Figure S2). To identify the bacterial taxa that were more abundant (in a statistically significant manner) in the patient group than in the healthy-adult group, *p*-values were calculated for all the taxa detected. The result demonstrated that distinct bacterial genera were overrepresented in the patient and healthy-adult groups. Whereas *Haemophilus* (p = 0.010) and *Moraxella* (p = 0.028) were identified as patient group-specific genera, *Streptococcus* (p = 0.003)*, Neisseria* (p = 0.003), *Gemella* (p = 0.003), *Aggregatibacter* (p = 0.008), and *Actinobacillus* (p = 0.001) were determined to be bacteria specific to the healthy-adult group.

### Oropharyngeal microbiome types

**Figure 3:**
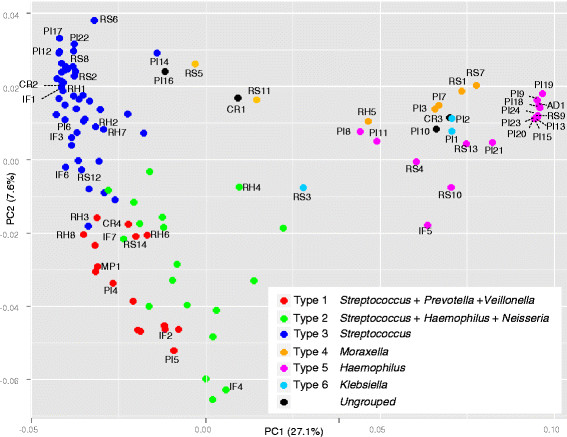
**Dependence of microbiome structure on several key genera.** Principal coordinate analysis (PCoA) of the bacterial communities isolated from 57 healthy-adult and 59 patient samples was performed using the weighted pairwise UniFrac distance matrix. The UniFrac distance represents the distance between 2 samples in terms of the microbial community structure. Samples are color-coded according to the 7 clustering groups.

To hierarchically visualize the bacterial profile similarities among the samples, a UPGMA dendrogram was generated from the Fast UniFrac distance matrix. Based on bacterial composition, the samples analyzed in this study were divided into 6 oropharyngeal microbiome types (Additional file 3: Figure S2), with the clusters being characterized by the dominance of several bacterial genera: Type 1 (dominated by *Streptococcus* + *Prevotella* + *Veillonella*), Type 2 (*Streptococcus* + *Haemophilus* + *Neisseria*), Type 3 (*Streptococcus*), Type 4 (*Moraxella*), Type 5 (*Haemophilus*), and Type 6 (*Klebsiella*). Only 4 samples were not grouped into any of the 6 types. The healthy adults and a subset of patients harbored bacterial communities dominated by *Streptococcus*, and to a lesser extent by *Haemophilus*, *Neisseria*, *Prevotella*, *Veillonella*, and/or *Gemella* (Types 1–3 in Figure 3). The remaining patients carried impaired microbiota dominated by *Moraxella* (Type 4 in Figure 3), *Haemophilus* (Type 5), or *Klebsiella* (Type 6), and this was coupled with a massive reduction in the levels of *Streptococcus*. Types 5 and 6 were dominated by well-known pathogens like *H. influenzae* and *K. pneumonia*, but Type 4 was dominated by a previously unknown one, *Moraxella nonliquefaciens*.

### Effect of causal virus type and other variables on microbiota

We elucidated the differences in bacterial profiles in the context of causal agents of infections and demonstrated that virus type did not determine the structural differences in bacterial communities (Additional file 4: Figure S3A). Moreover, sex, sample type (swab, aspirate, or sputum), and smoking status did not influence the bacterial community structure (Additional file 4: Figures S3B, S3C and S3D), which was also unaffected by the year and month of sample collection (data not shown). By contrast, subjects’ age was associated with the bacterial profile in a statistically significant manner (Additional file 4: Figure S3E), and the samples were categorized into 2 age groups, 0-5 and 6-90 years (*p* <0.0001, Hotelling’s test).

### *Moraxella nonliquefaciens* and *M. catarrhalis*

In this study, we discovered a bacterial species that was dominant in young patients (0-5 years old): *M. nonliquefaciens* was detected in 32.2% of the patients, with abundance ratios of 0.03%-97.0% depending on the subject, but this species was not observed in any healthy subject. Most of the patients (16 out of 19 cases) harboring *M. nonliquefaciens* were under 6 years old. Two RSV infected patients (RS1 and RS7) showed 95 and 97% of abundance of *M. nonliquefaciens*, indicating that the upper respiratory tracts of these patients were overwhelmed by this bacterial species. In addition, a closely related pathogenic species, *M. catarrhalis,* was detected in 23.7% of the patients, with abundance ratios of 0.03%-26.5%.

**Figure 4:**
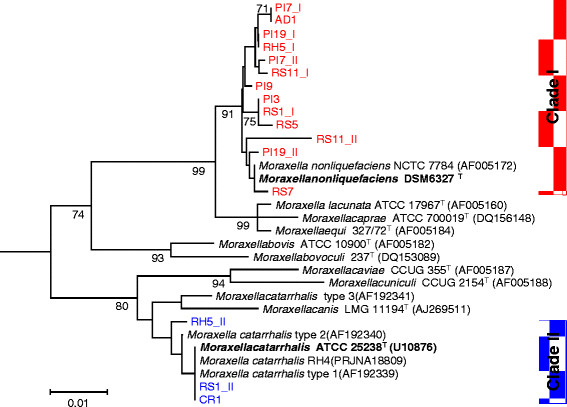
**Phylogenetic tree based on 16S rRNA gene sequences showing the relationship of the newly discovered*Moraxella*sequences with publically available sequences of other*Moraxella*strains.** Representatives of 3*M. catarrhalis* type strains were included together with the type strains of *Moraxella* species. Red text and blue test indicate *M. nonliquefaciens* contigs and *M. catarrhalis* contigs recovered from patient samples, respectively. The neighbor-joining tree was evaluated using 1,000 bootstrap pseudoreplicates. Only bootstrap values over 70% are shown at branch nodes. The scale bar represents the genetic distance.

Analyzing the 16S rRNA gene sequence revealed that *M. nonliquefaciens* and *M. catarrhalis* were clearly distinct species that showed 98.4% similarity between type strains (Additional file 5: Figure S4). The *Moraxella* contigs recovered from patient samples were divided into 2 clades based on phylogenetic analysis (Figure 4). Clade I was closely related (98.5%-99.8% 16S rRNA gene similarity) to the type strain of *M. nonliquefaciens*. The branching pattern of the contigs within the radiation of Clade I indicated that *M. nonliquefaciens* populations in the patients encompassed 3 phylogenetic lineages. Contigs belonging to Clade II were clustered together with *M. catarrhalis* (99.6%-100% 16S rRNA gene similarity), and these contigs were further divided into 2 subpopulations, Type 1 and Type 2 populations [29].

### Evaluating potential pathogenicity of M. nonliquefaciens based on genome sequences

To determine whether *M. nonliquefaciens* has a possibility to be an opportunistic pathogen, its potential pathogenicity was inferred using the genome sequence of the type strain. Genome sequencing identified a 2.22-Mb-sized genome of *M. nonliquefaciens* DSM 6327^T^, featuring a 42.06% G + C ratio. The genomic relatedness calculated using ANI showed that *M. nonliquefaciens* DSM 6327^T^ and *M. catarrhalis* RH4 shared low genomic relatedness, 75% ANI, which is considerably less than the cut-off value used for species circumscription, 95%-96% ANI [30]. The ANI value further confirmed that the 2 species were distinct.

Various proteins have been reported to play pivotal roles in *M. catarrhalis* pathogenesis [31]. Thus, we examined whether the virulence proteins in the *M. catarrhalis* RH4 genome were also encoded in the newly determined *M. nonliquefaciens* genome*.* The genes responsible for host-cell adhesion and invasion, evasion of host immune system, and biofilm formation were included as putative virulence factors. Comparative genomic analysis revealed that most of the virulence genes identified in *M. catarrhalis* were encoded in the *M. nonliquefaciens* genome (Additional file 6: Table S2). Moreover, resistance to *β*-lactam antibiotics was predicted based on the presence of the *β*-lactamase Class C gene. However, BRO-1 and BRO-2 *β-*lactamases encoded by most of *M. catarrhalis* strains were not detected in *M. nonliquefaciens.*

## Discussion

Our results demonstrated that changes in bacterial profiles elicited by viral infection were not associated with the causal viral species: the microbiome compositions in samples obtained from various viral infections were not differentiated based on the causal infectious agents. Regardless of the causal agents involved, the respiratory tract microbiota of patients differed substantially from the microbiota of healthy subjects in the kinds and diversities of prevalent bacteria. However, the heterogenicity of the patient group of this study (age, sampling type, and number of samples/virus type) hinders strong conclusions for this point. Nevertheless, the current results from this study provide the first insight into microbiome alterations associated with viral infection in the upper respiratory tract.

The diminished bacterial diversity observed in patients agreed well with previous studies reporting that the diversity in commensal airway microbiota declined following infection by specific pathogens [6],[13]. For example, in the nares of patients with *S. aureus* carriage, species diversity was half of that found in healthy adults’ nares [6]. These results indicate that the normal microflora is depleted in respiratory tract cells impaired due to viral infection and is replaced by a few opportunistic pathogens. Moreover, the dominance of *Streptococcus* in the respiratory tracts of healthy subjects (Table 1) agreed with previous culture-independent massive metagenomic sequencing studies [3],[7]. *Streptococcus* is also known to be abundant in the oral cavity [32],[33]. Thus, the oropharynx of healthy people could be characterized by high bacterial diversity and by an overwhelming abundance of the genus *Streptococcus.*

By analyzing the bacterial community, we defined 6 oropharyngeal types of bacterial populations in the upper respiratory tract. We use the word “oropharyngeal type” here based on the “enterotype” concept, which was introduced by Arumugam *et al.* and defined as the clusters of human gut microbiome determined based on bacterial composition [34]. The concept suggests that people can be classified into several enterotypes according to the abundance of key bacterial taxa in gut microbial communities [35]. In this study, the samples included were restricted to one ethnic group, and thus the suggested 6 oropharyngeal types may be accepted only temporarily. However, because no other efforts to cluster respiratory tract microbiomes have been reported to date, our results may serve as a favorable starting point for future studies on this subject.

Our results revealed that *Haemophilus* and *Moraxella* were patient-specific genera. Unlike *H. influenzae*, *M. nonliquefaciens* has not been studied for its possible role in pneumonia. Although *M. nonliquefaciens* has been isolated from clinical cases including chronic bronchitis [36], bronchial infection [37], pneumonitis [38], endophthalmitis [39], septic arthritis [40], thyroiditis [41], discitis [42], botryomycosis [43], and endocarditis [44], this bacterium is widely considered to be a part of the normal flora in the human upper respiratory tract and to exhibit low pathogenicity [45],[46]. By contrast, a closely related species, *M. catarrhalis*, is a well-studied respiratory tract pathogen that frequently colonizes the nasopharynx and is an exclusively human pathogen that displays an affinity for the human upper respiratory tract [47],[48]. Long considered to be a commensal bacterium of the upper respiratory tract, *M. catarrhalis* has now been established as an etiological cause of otitis media and the exacerbations of chronic obstructive pulmonary disease (COPD) [47],[49].

Despite the distinctiveness of the 2 species at the taxonomic level, *M. nonliquefaciens* and *M. catarrhalis* share several common features. First, the age-related incidence of *M. nonliquefaciens* infection determined here is concordant with that of *M. catarrhalis.* Previously, *M. catarrhalis* was reported to be mostly associated with upper respiratory tract infections in children [50], and its carriage rate was shown to be high in children (up to 75%) and extremely low in healthy adults (1%–3%) [49],[51]-[54]. Moreover, the phenotype and gene incidences of *M. catarrhalis* isolates of children and adults presenting with respiratory disease differ substantially, possibly as a result of immune evasion in adults [48]. The age-related incidence of *M. nonliquefaciens* and *M. catarrhalis* may be indicative of the weak pathogenicity of *Moraxella* species, which may be unable to evade the well-established immune system of adults.

Second, most strains of *M. catarrhalis* are known to produce *β-*lactamases and thus exhibit ampicillin resistance [49]. This antibiotic resistance was also predicted in *M. nonliquefaciens* based on the presence of the Class C *β-*lactamase gene. However, although both species possess Class C *β-*lactamase genes, the species differ with respect to the possession of BRO *β-*lactamase; BRO is unique because it shows no substantial similarity to any *β-*lactamase genes identified so far [55]. The absence of BRO-1 and BRO-2 in *M. nonliquefaciens* suggests that *M. catarrhalis* acquired the BRO genes by means of lateral gene transfer after the 2 species evolved into distinct lineages.

Third, all but one virulence factors reported for *M. catarrhalis* were found to be encoded by *M. nonliquefaciens* (Additional file 6: Table S2), which indicates that *M. nonliquefaciens* has a high potential to be pathogenic even though it is currently considered to be a commensal bacterium. Several reasons may account for why the overabundance of *M. nonliquefaciens* has not been reported. Growing this organism and distinguishing it from *M. catarrhalis* are challenging, which may have resulted in a poor recognition of *M. nonliquefaciens* as a respiratory pathogen. Moreover, although *M. catarrhalis* is focused on by clinicians, the isolation of *M. catarrhalis* from clinical samples is complicated by the presence of *Neisseria* strains because these organisms share morphological similarities [49]. Furthermore, *M. nonliquefaciens* may have been considerably underestimated because of being misidentified as *M. catarrhalis* or *Neisseria* spp. [49]. Lastly, the current absence of clinical interest or familiarity with *M. nonliquefaciens* may have resulted in under-reporting or identification of this pathogen. Indeed, *M. catarrhalis* was previously underreported since other better recognized pathogens were also recognized and growing in the same cultures [49]. To clarify the incidence of this potential pathogen, future studies will need to differentiate between the true rates of incidence of *M. catarrhalis* and *M. nonliquefaciens.* The results of this study suggest that *M. nonliquefaciens* deserves considerable attention as a potential opportunistic pathogen in the respiratory tract.

## Conclusions

The pyrosequencing of bacterial community identified 6 oropharyngeal microbiome types in the upper respiratory tract, but the bacterial profile was not related to the type of causal infected viruses. The microbiota of patients differed substantially from that of healthy subjects in the kinds and diversities of prevalent bacteria. Comparative analysis of healthy adults and patients identified a bacterium specific to young patients, *M. nonliquefaciens.* The results of whole-genome sequencing raised the possibility of *M. nonliquefaciens* being an opportunistic pathogen.

## Authors’ contributions

HY, DY and JC conceived and designed research. DY and KL collected samples and clinical data. HY, YC and DY performed research. HY, DY, YC and JC analyzed data. HY, DY and JC wrote the paper. All authors read and approved the final manuscript.

## Additional files

## Electronic supplementary material

Additional file 1: Table S1.: List and clinical characteristics of samples used in this study. ND, not determined. (DOCX 27 KB)

Additional file 2: Figure S1.: Ordination diagram showing the relatedness of microbiomes in the upper respiratory tract of healthy people. Principal coordinate analysis (PCoA) of bacterial communities isolated from 57 healthy adults was performed using the weighted pairwise UniFrac distance matrix. The UniFrac distance represents the distance between 2 samples in terms of the microbial community structure. (PPTX 97 KB)

Additional file 3: Figure S2.: Dendrogram and circle map showing the clustering of samples into 6 groups depending on bacterial population dynamics. The unweighted pair group method with arithmetic mean (UPGMA) dendrogram was generated from the Fast UniFrac distance matrix to hierarchically visualize the manner in which samples are grouped. The relative abundance of representative microbial genera is indicated as a circle map; circle sizes represent the percentage ratio within a sample. (PPTX 190 KB)

Additional file 4: Figure S3.: Ordination diagram showing the relatedness of microbiomes in the upper respiratory tract of people afflicted with diverse viral infections. We performed principal coordinate analysis (PCoA) on the bacterial communities isolated from 57 healthy-adult and 59 patient samples by using the weighted pairwise UniFrac distance matrix. The UniFrac distance represents the distance between 2 samples in terms of the microbial community structure. The structure of the bacterial community was not affected by (A) the type of virus or by (B) the sex, (C) sample type, and (D) smoking status of the subjects. However, (E) age was correlated with the microbiome structure. (PPTX 230 KB)

Additional file 5: Figure S4.: Diagram showing the relationships among the 2 type strains and the 2 representative contigs obtained from patient samples. The average nucleotide identity (ANI) value indicating genome relatedness was calculated using the complete genome sequence of *M. catarrhalis* RH4 strain (PRJNA 48809), which shows 100% 16S rRNA gene sequence identity with the type strain of the species. (PPTX 45 KB)

Additional file 6: Table S2.: Comparison of major virulence genes present in *M. nonliquefaciens* and *M. catarrhalis.* The list of virulent genes was obtained from previous reports [31],[56]. The gene locus in each genome is presented together with gene size in amino acids (in parenthesis). (DOCX 22 KB)

Below are the links to the authors’ original submitted files for images.

Authors’ original file for figure 1

Authors’ original file for figure 2

Authors’ original file for figure 3

Authors’ original file for figure 4

## Abbreviations

UPGMA: Unweighted pair group method with arithmetic mean
PCoA: Principal coordinate analysis
ANI: Average nucleotide identity
VTM: Viral transport medium
ICU: Intensive care unit
