# Supplementary material for: Profiling bacterial community in upper respiratory tracts
Source: BMC Infect Dis. 2014 Nov 13;14:583. doi: 10.1186/s12879-014-0583-3 (PMC4236460; doi:10.1186/s12879-014-0583-3)
Supplement: Supplementary file 1 — Additional file 1: Table S1.: List and clinical characteristics of samples used in this study. ND, not determined. (DOCX 27 KB) [file 12879_2014_583_MOESM1_ESM.docx]

**TABLE S1** List and clinical characteristics of samples used in this study. ND, not determined.

| **ID** | **Sampling date** | **Sex** | **Age** | **Sample type** | **Smoking** |
| --- | --- | --- | --- | --- | --- |
| **Hos1** | 2011-06-21 | F | 52 | swab | no |
| **Hos2** | 2011-06-21 | F | 45 | swab | no |
| **Hos3** | 2011-06-21 | M | 43 | swab | no |
| **Hos4** | 2011-06-21 | M | 58 | swab | no |
| **Hos5** | 2011-06-21 | M | 26 | swab | no |
| **Hos6** | 2011-06-21 | M | 30 | swab | no |
| **Hos7** | 2011-06-21 | F | 27 | swab | no |
| **Hos8** | 2011-06-21 | F | 38 | swab | no |
| **Hos9** | 2011-06-21 | M | 47 | swab | no |
| **ICU1** | 2011-06-30 | F | 36 | swab | no |
| **ICU2** | 2011-06-30 | F | 30 | swab | no |
| **ICU3** | 2011-06-30 | F | 36 | swab | no |
| **ICU4** | 2011-06-30 | F | 50 | swab | no |
| **ICU5** | 2011-06-30 | F | 43 | swab | no |
| **ICU6** | 2011-06-30 | F | 41 | swab | no |
| **ICU7** | 2011-06-30 | F | 35 | swab | no |
| **ICU8** | 2011-06-30 | F | 32 | swab | no |
| **Com1** | 2011-06-12 | F | 30 | swab | no |
| **Com2** | 2011-06-12 | M | 22 | swab | no |
| **Com3** | 2011-06-12 | F | 27 | swab | no |
| **Com4** | 2011-06-12 | F | 54 | swab | no |
| **Com5** | 2011-06-12 | M | 43 | swab | no |
| **Com6** | 2011-06-12 | F | 40 | swab | no |
| **Com7** | 2011-06-12 | F | 35 | swab | no |
| **Com8** | 2013-06-02 | M | 38 | swab | no |
| **Com9** | 2013-06-02 | F | 48 | swab | no |
| **Com10** | 2013-06-02 | F | 29 | swab | no |
| **Com11** | 2013-06-02 | F | 30 | swab | no |
| **Com12** | 2013-06-02 | F | 28 | swab | no |
| **Com13** | 2013-06-02 | F | 24 | swab | no |
| **Com14** | 2013-06-02 | M | 25 | swab | no |
| **Com15** | 2013-06-02 | M | 30 | swab | no |
| **Com16** | 2013-06-02 | F | 33 | swab | no |
| **Com17** | 2013-06-02 | F | 25 | swab | no |
| **Com18** | 2013-06-02 | M | 21 | swab | no |
| **Com19** | 2013-06-02 | M | 27 | swab | no |
| **Com20** | 2013-06-02 | M | 35 | swab | no |
| **Com21** | 2013-06-13 | F | 24 | swab | no |
| **Com22** | 2013-06-13 | F | 26 | swab | no |
| **Com23** | 2013-06-13 | F | 26 | swab | no |
| **Com24** | 2013-06-13 | M | 36 | swab | no |
| **Com25** | 2013-06-13 | F | 27 | swab | no |
| **Com26** | 2013-06-13 | F | 24 | swab | no |
| **Com27** | 2013-06-13 | M | 30 | swab | yes |
| **Com28** | 2013-06-13 | M | 31 | swab | yes |
| **Com29** | 2013-06-13 | M | 28 | swab | yes |
| **Com30** | 2013-06-13 | M | 27 | swab | no |
| **Com31** | 2013-06-13 | M | 34 | swab | yes |
| **Com32** | 2013-06-13 | F | 31 | swab | no |
| **Com33** | 2013-06-13 | F | 30 | swab | no |
| **Com34** | 2013-06-13 | M | 50 | swab | yes |
| **Com35** | 2013-06-13 | F | 54 | swab | no |
| **Com36** | 2013-06-13 | F | 30 | swab | no |
| **Com37** | 2013-06-13 | M | 24 | swab | no |
| **Com38** | 2013-06-13 | M | 46 | swab | yes |
| **Com39** | 2013-06-13 | F | 24 | swab | no |
| **Com40** | 2013-06-13 | F | 45 | swab | no |
| **IF1** | 2010-12-07 | M | 1 | swab | ND |
| **IF2** | 2010-12-13 | M | 9 | swab | ND |
| **IF3** | 2010-12-31 | M | 2 | swab | ND |
| **IF4** | 2011-01-27 | M | 59 | swab | no |
| **IF5** | 2013-01-08 | M | 62 | sputum | no |
| **IF6** | 2013-01-10 | F | 32 | sputum | no |
| **IF7** | 2013-01-19 | M | 13 | aspirate | no |
| **PI1** | 2011-05-17 | F | 4 | swab | ND |
| **PI2** | 2011-05-18 | F | 0 | swab | ND |
| **PI3** | 2011-05-25 | F | 1 | swab | ND |
| **PI4** | 2011-05-30 | F | 3 | swab | ND |
| **PI5** | 2011-05-31 | M | 0 | swab | ND |
| **PI6** | 2011-06-07 | F | 1 | swab | ND |
| **PI7** | 2011-05-18 | F | 4 | swab | ND |
| **PI8** | 2012-05-02 | M | 1 | swab | ND |
| **PI9** | 2012-05-02 | F | 1 | swab | ND |
| **PI10** | 2012-05-02 | M | 3 | swab | ND |
| **PI11** | 2012-05-07 | F | 1 | swab | ND |
| **PI12** | 2012-05-07 | F | 2 | swab | ND |
| **PI13** | 2012-05-09 | F | 1 | swab | ND |
| **PI14** | 2012-05-10 | F | 0 | swab | ND |
| **PI15** | 2012-05-14 | F | 2 | swab | ND |
| **PI16** | 2012-05-14 | F | 4 | swab | ND |
| **PI17** | 2012-05-15 | F | 1 | swab | ND |
| **PI18** | 2012-05-15 | M | 1 | swab | ND |
| **PI19** | 2012-05-15 | F | 1 | swab | ND |
| **PI20** | 2012-05-17 | M | 2 | swab | ND |
| **PI21** | 2012-05-17 | M | 2 | swab | ND |
| **PI22** | 2013-04-22 | F | 0 | aspirate | ND |
| **PI23** | 2013-04-25 | M | 2 | aspirate | ND |
| **PI24** | 2013-05-04 | M | 0 | aspirate | ND |
| **RH1** | 2012-06-29 | M | 78 | sputum | no |
| **RH2** | 2012-06-07 | M | 59 | sputum | yes |
| **RH3** | 2012-06-18 | M | 40 | aspirate | yes |
| **RH4** | 2012-12-20 | M | 58 | sputum | yes |
| **RH5** | 2012-12-22 | M | 0 | aspirate | ND |
| **RH6** | 2013-01-03 | F | 67 | sputum | no |
| **RH7** | 2013-01-05 | M | 0 | aspirate | ND |
| **RH8** | 2013-01-11 | M | 1 | aspirate | ND |
| **RS1** | 2011-01-17 | F | 1 | swab | ND |
| **RS2** | 2011-02-10 | F | 0 | swab | ND |
| **RS3** | 2011-02-14 | M | 1 | swab | ND |
| **RS4** | 2011-02-28 | M | 1 | swab | ND |
| **RS5** | 2011-05-13 | M | 2 | swab | ND |
| **RS6** | 2012-12-15 | M | 1 | aspirate | ND |
| **RS7** | 2012-12-19 | F | 0 | aspirate | ND |
| **RS8** | 2012-12-19 | F | 0 | aspirate | ND |
| **RS9** | 2012-12-24 | F | 2 | aspirate | ND |
| **RS10** | 2012-12-25 | F | 2 | aspirate | ND |
| **RS11** | 2012-12-27 | F | 2 | sputum | ND |
| **RS12** | 2012-12-31 | F | 2 | sputum | ND |
| **RS13** | 2013-01-02 | M | 0 | aspirate | ND |
| **RS14** | 2013-01-02 | F | 71 | sputum | no |
| **CR1** | 2012-12-23 | M | 0 | aspirate | ND |
| **CR2** | 2012-12-27 | F | 88 | aspirate | no |
| **CR3** | 2013-01-20 | F | 66 | sputum | no |
| **CR4** | 2013-01-21 | F | 80 | sputum | no |
| **AD1** | 2012-12-26 | M | 0 | aspirate | ND |
| **MP1** | 2013-02-06 | M | 75 | sputum | ND |
