# Supplementary material for: Profiling bacterial community in upper respiratory tracts
Source: BMC Infect Dis. 2014 Nov 13;14:583. doi: 10.1186/s12879-014-0583-3 (PMC4236460; doi:10.1186/s12879-014-0583-3)
Supplement: Supplementary file 2 — Additional file 2: Figure S1.: Ordination diagram showing the relatedness of microbiomes in the upper respiratory tract of healthy people. Principal coordinate analysis (PCoA) of bacterial communities isolated from 57 healthy adults was performed using the weighted pairwise UniFrac distance matrix. The UniFrac distance represents the distance between 2 samples in terms of the microbial community structure. (PPTX 97 KB) [file 12879_2014_583_MOESM2_ESM.pptx]

## Slide 1
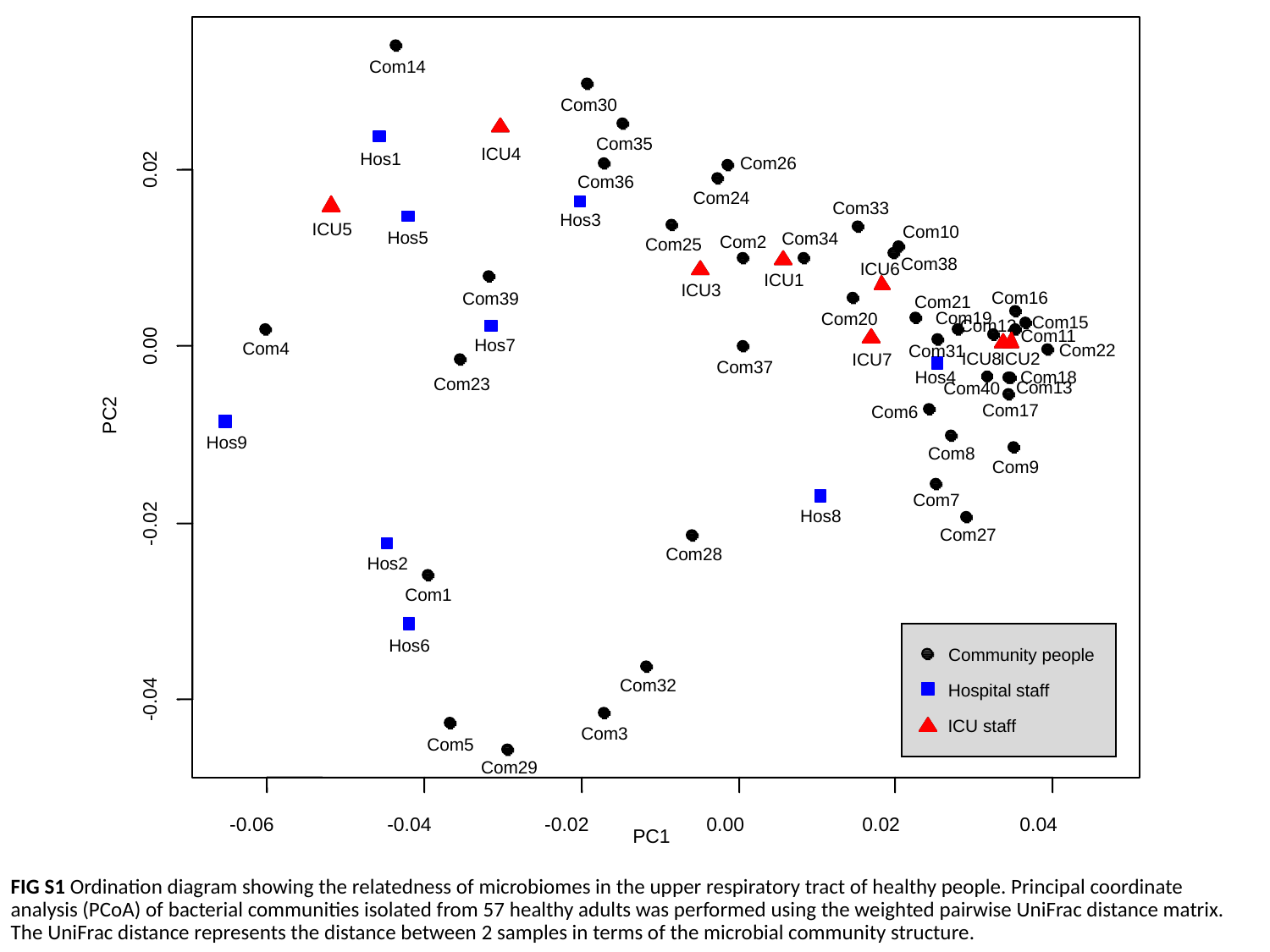

Com14
Com30
Com35
ICU4
Hos1
Com26
0.02
Com36
Com24
Com33
Hos3
ICU5
Com10
Hos5
Com34
Com2
Com25
Com38
ICU6
ICU1
ICU3
Com16
Com39
Com21
Com19
Com20
Com15
Com12
Com11
Hos7
0.00
Com4
Com22
Com31
ICU8
ICU2
ICU7
Com37
Com18
Hos4
Com23
Com13
Com40
Com17
Com6
PC2
Hos9
Com8
Com9
Com7
Hos8
-0.02
Com27
Com28
Hos2
Com1
Hos6
Community people
Com32
Hospital staff
-0.04
ICU staff
Com3
Com5
Com29
-0.06
-0.04
-0.02
0.00
0.02
0.04
PC1
FIG S1 Ordination diagram showing the relatedness of microbiomes in the upper respiratory tract of healthy people. Principal coordinate analysis (PCoA) of bacterial communities isolated from 57 healthy adults was performed using the weighted pairwise UniFrac distance matrix. The UniFrac distance represents the distance between 2 samples in terms of the microbial community structure.
