# Supplementary material for: Profiling bacterial community in upper respiratory tracts
Source: BMC Infect Dis. 2014 Nov 13;14:583. doi: 10.1186/s12879-014-0583-3 (PMC4236460; doi:10.1186/s12879-014-0583-3)
Supplement: Supplementary file 3 — Additional file 3: Figure S2.: Dendrogram and circle map showing the clustering of samples into 6 groups depending on bacterial population dynamics. The unweighted pair group method with arithmetic mean (UPGMA) dendrogram was generated from the Fast UniFrac distance matrix to hierarchically visualize the manner in which samples are grouped. The relative abundance of representative microbial genera is indicated as a circle map; circle sizes represent the percentage ratio within a sample. (PPTX 190 KB) [file 12879_2014_583_MOESM3_ESM.pptx]

## Slide 1
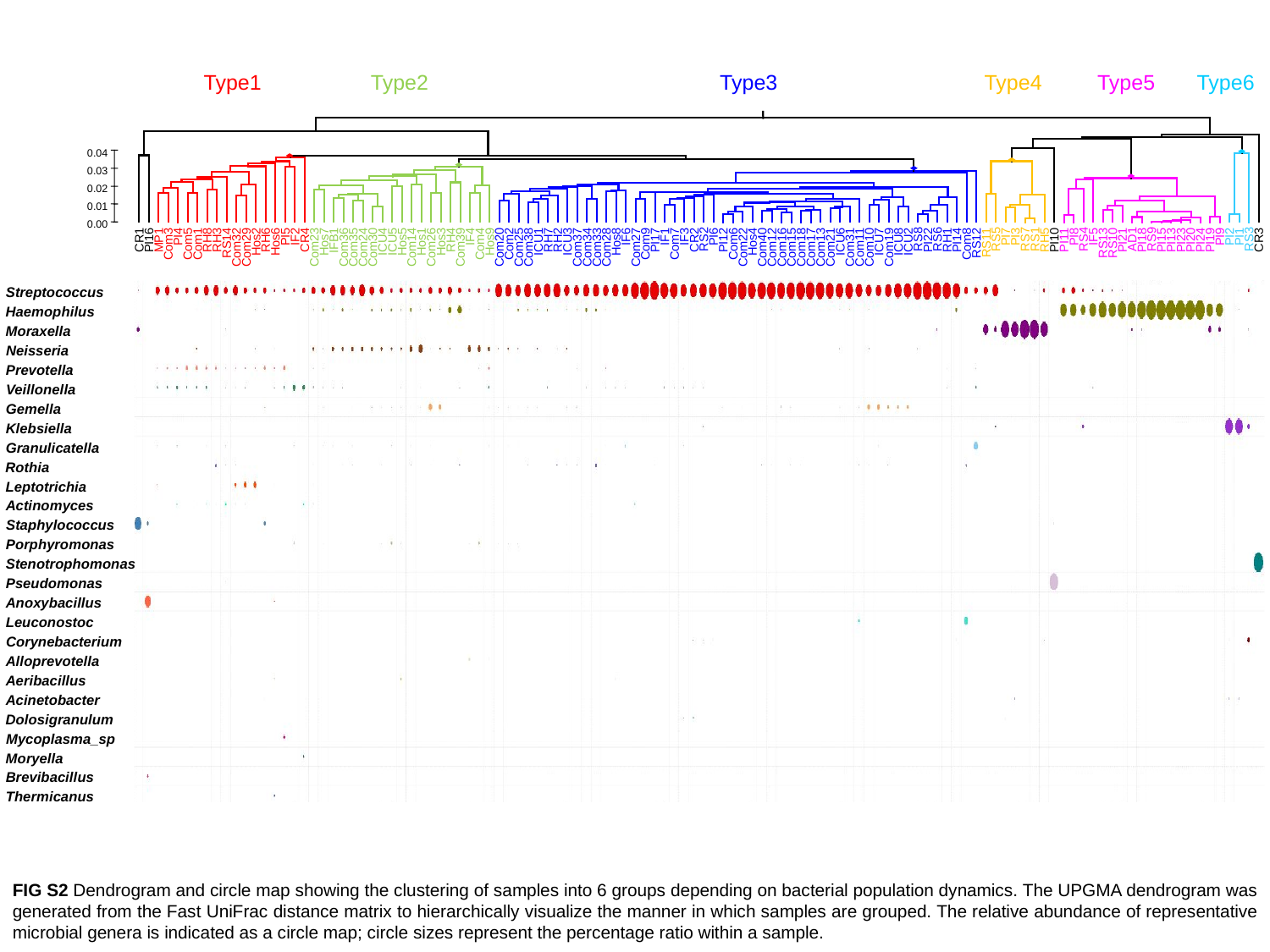

Type1
Type2
Type3
Type4
Type5
Type6
0.04
0.03
0.02
0.01
0.00
 IF6
 IF1
 IF3
 IF2
 IF4
 IF5
 PI4
 PI6
 PI9
 PI2
 PI1
 PI5
 PI7
 PI3
 PI8
 RS2
 RS3
 RS8
 RS6
 RS5
 RS7
 RS1
 RS4
 AD1
 RS9
 CR1
 RH8
 CR2
 CR3
 RH3
 RH6
 CR4
 RH4
 RH7
 RH2
 RH1
 RH5
 PI16
 MP1
 PI17
 PI12
 PI15
 PI13
 PI23
 PI20
 PI24
 PI19
 PI22
 PI14
 PI10
 PI11
 PI21
 PI18
 IFB1
 ICU4
 ICU5
 ICU1
 ICU3
 ICU6
 ICU7
 ICU8
 ICU2
 Hos8
 Hos4
 Hos2
 Hos6
 Hos7
 Hos5
 Hos1
 Hos3
 Hos9
 RS14
 RS12
 RS11
 RS13
 RS10
 Com3
 Com5
 Com1
 Com9
 Com7
 Com6
 Com4
 Com2
 Com8
 Com32
 Com29
 Com23
 Com36
 Com35
 Com24
 Com30
 Com14
 Com26
 Com39
 Com20
 Com25
 Com38
 Com37
 Com34
 Com33
 Com28
 Com27
 Com22
 Com40
 Com12
 Com16
 Com15
 Com18
 Com17
 Com13
 Com21
 Com31
 Com11
 Com10
 Com19
Streptococcus
Haemophilus
Moraxella
Neisseria
Prevotella
Veillonella
Gemella
Klebsiella
Granulicatella
Rothia
Leptotrichia
Actinomyces
Staphylococcus
Porphyromonas
Stenotrophomonas
Pseudomonas
Anoxybacillus
Leuconostoc
Corynebacterium
Alloprevotella
Aeribacillus
Acinetobacter
Dolosigranulum
Mycoplasma_sp
Moryella
Brevibacillus
Thermicanus
FIG S2 Dendrogram and circle map showing the clustering of samples into 6 groups depending on bacterial population dynamics. The UPGMA dendrogram was generated from the Fast UniFrac distance matrix to hierarchically visualize the manner in which samples are grouped. The relative abundance of representative microbial genera is indicated as a circle map; circle sizes represent the percentage ratio within a sample.
