# Supplementary material for: Profiling bacterial community in upper respiratory tracts
Source: BMC Infect Dis. 2014 Nov 13;14:583. doi: 10.1186/s12879-014-0583-3 (PMC4236460; doi:10.1186/s12879-014-0583-3)
Supplement: Supplementary file 4 — Additional file 4: Figure S3.: Ordination diagram showing the relatedness of microbiomes in the upper respiratory tract of people afflicted with diverse viral infections. We performed principal coordinate analysis (PCoA) on the bacterial communities isolated from 57 healthy-adult and 59 patient samples by using the weighted pairwise UniFrac distance matrix. The UniFrac distance represents the distance between 2 samples in terms of the microbial community structure. The structure of the bacterial community was not affected by (A) the type of virus or by (B) the sex, (C) sample type, and (D) smoking status of the subjects. However, (E) age was correlated with the microbiome structure. (PPTX 230 KB) [file 12879_2014_583_MOESM4_ESM.pptx]

## Slide 1
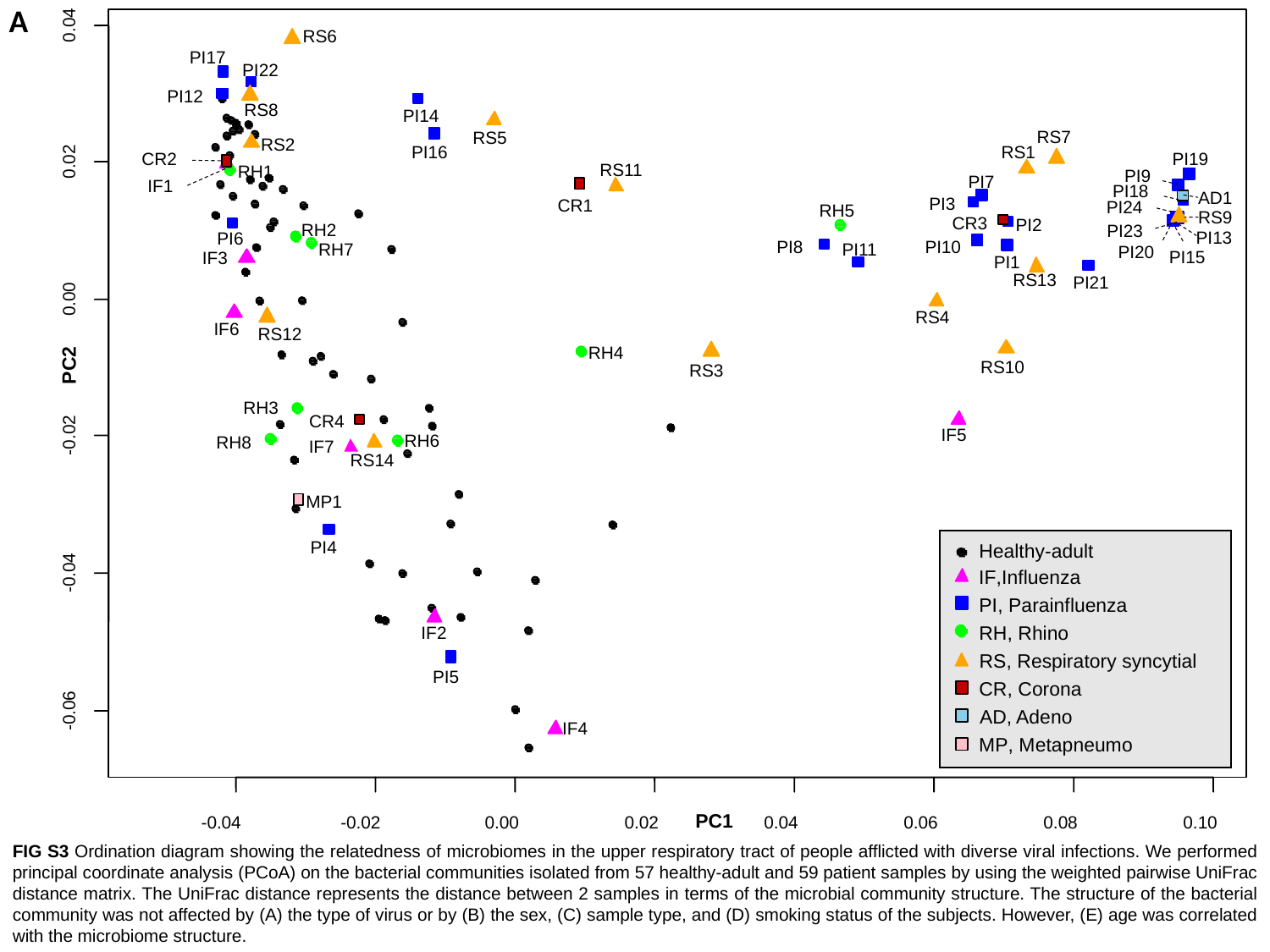

A
0.04
RS6
PI17
PI22
PI12
RS8
PI14
RS7
RS5
RS2
PI16
RS1
CR2
PI19
0.02
RS11
RH1
PI9
PI7
IF1
PI18
AD1
PI3
CR1
PI24
RH5
RS9
CR3
PI2
RH2
PI23
PI13
PI6
PI10
PI8
RH7
PI11
PI20
PI15
IF3
PI1
RS13
PI21
0.00
RS4
IF6
RH4
RS3
RH6
RS14
IF2
PI5
IF4
RS12
PC2
RS10
RH3
CR4
IF5
-0.02
RH8
IF7
MP1
PI4
Healthy-adult
-0.04
IF,Influenza
PI, Parainfluenza
RH, Rhino
RS, Respiratory syncytial
CR, Corona
-0.06
AD, Adeno
MP, Metapneumo
PC1
-0.04
-0.02
0.00
0.02
0.04
0.06
0.08
0.10
FIG S3 Ordination diagram showing the relatedness of microbiomes in the upper respiratory tract of people afflicted with diverse viral infections. We performed principal coordinate analysis (PCoA) on the bacterial communities isolated from 57 healthy-adult and 59 patient samples by using the weighted pairwise UniFrac distance matrix. The UniFrac distance represents the distance between 2 samples in terms of the microbial community structure. The structure of the bacterial community was not affected by (A) the type of virus or by (B) the sex, (C) sample type, and (D) smoking status of the subjects. However, (E) age was correlated with the microbiome structure.

## Slide 2
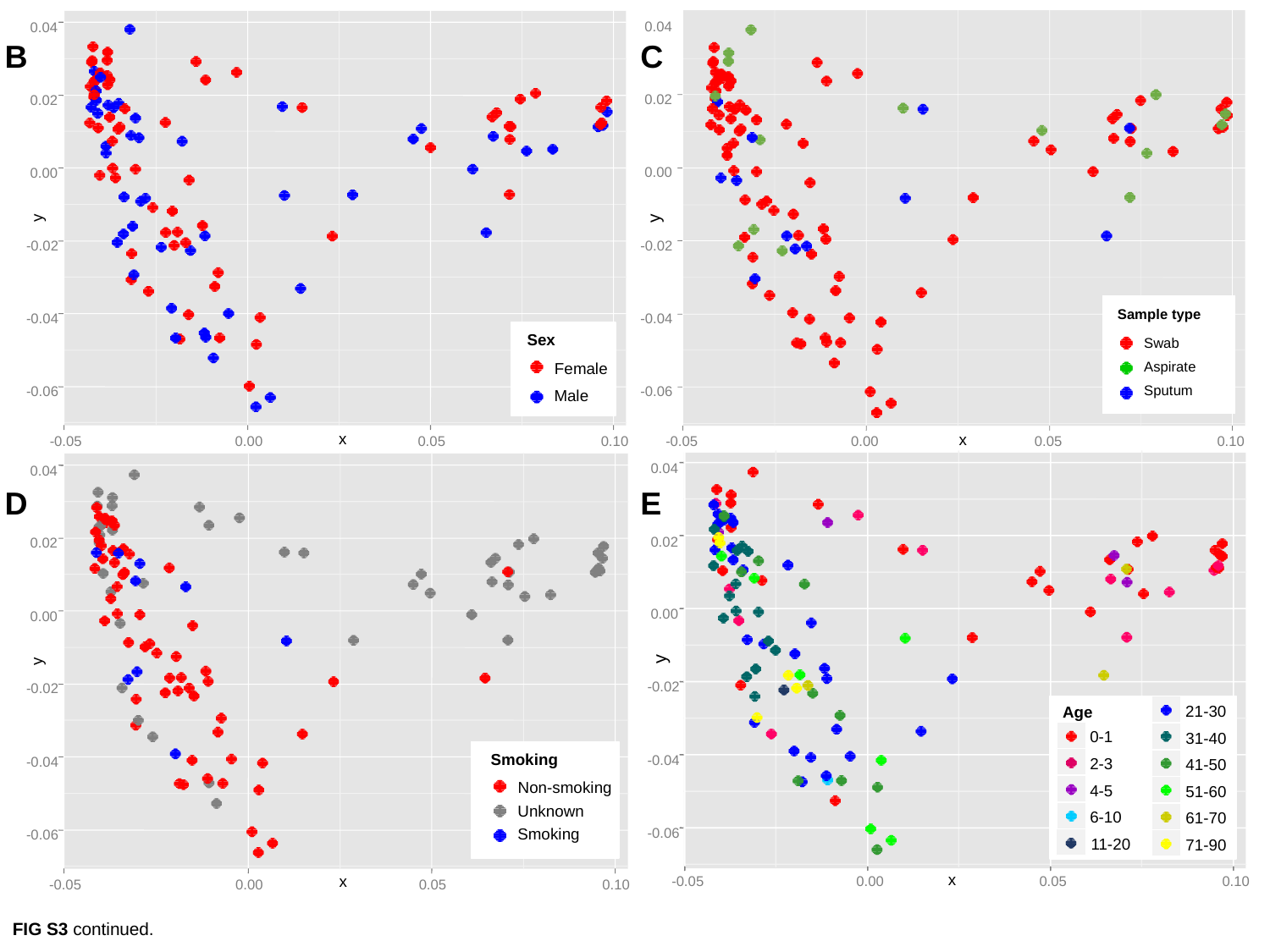

0.04
C
0.02
0.00
y
-0.02
Sample type
-0.04
Swab
Aspirate
Sputum
-0.06
x
-0.05
0.00
0.05
0.10
0.04
0.02
0.00
y
-0.02
-0.04
Sex
Female
Male
-0.06
x
-0.05
0.00
0.05
0.10
B
0.04
0.02
0.00
y
-0.02
21-30
Age
0-1
31-40
-0.04
2-3
41-50
4-5
51-60
6-10
61-70
-0.06
11-20
71-90
x
-0.05
0.00
0.05
0.10
0.04
0.02
0.00
y
-0.02
Smoking
Non-smoking
Unknown
Smoking
-0.04
-0.06
x
-0.05
0.00
0.05
0.10
D
E
FIG S3 continued.
