# Supplementary material for: Profiling bacterial community in upper respiratory tracts
Source: BMC Infect Dis. 2014 Nov 13;14:583. doi: 10.1186/s12879-014-0583-3 (PMC4236460; doi:10.1186/s12879-014-0583-3)
Supplement: Supplementary file 5 — Additional file 5: Figure S4.: Diagram showing the relationships among the 2 type strains and the 2 representative contigs obtained from patient samples. The average nucleotide identity (ANI) value indicating genome relatedness was calculated using the complete genome sequence of M. catarrhalis RH4 strain (PRJNA 48809), which shows 100% 16S rRNA gene sequence identity with the type strain of the species. (PPTX 45 KB) [file 12879_2014_583_MOESM5_ESM.pptx]

## Slide 1
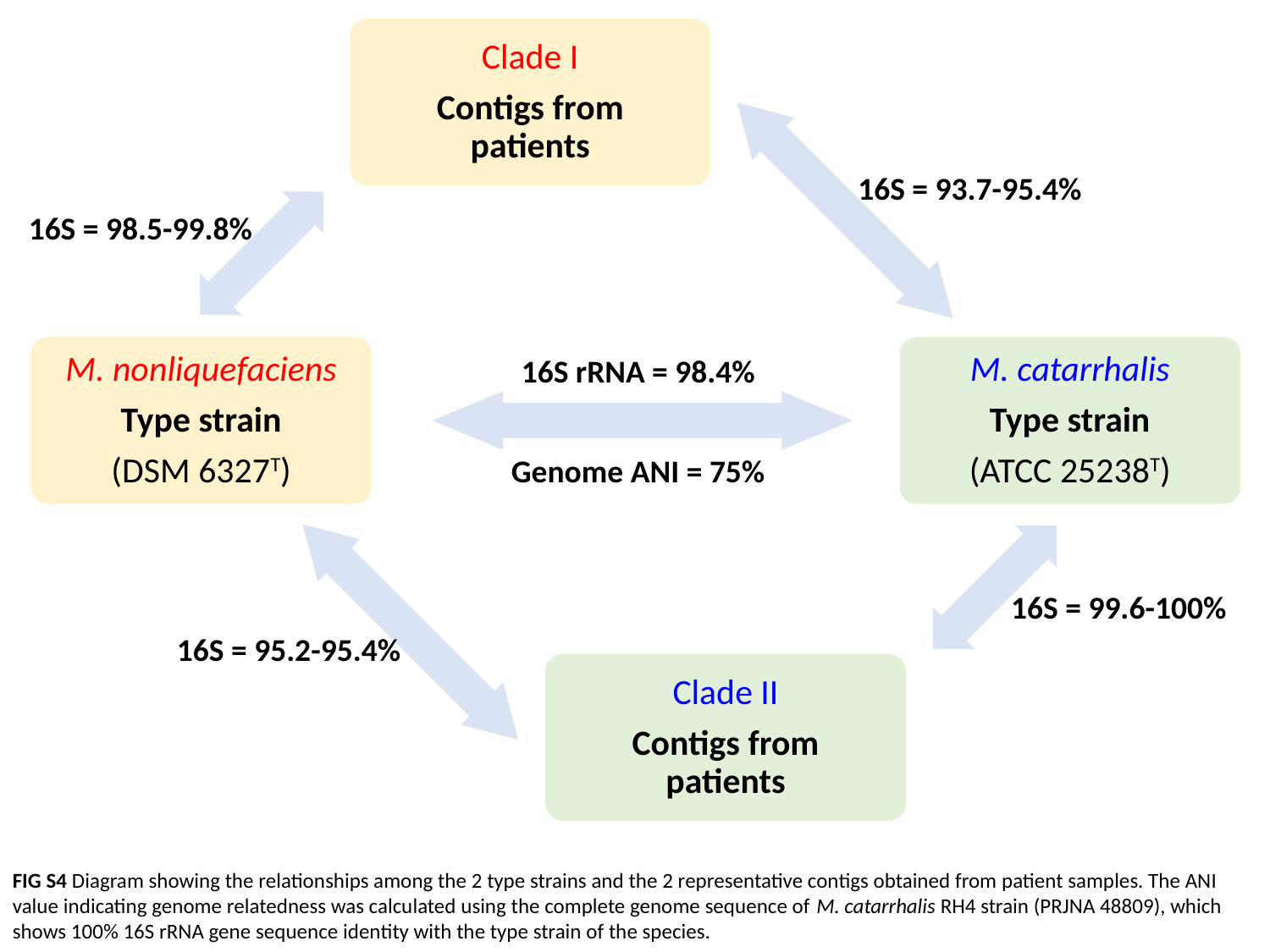

Clade I
Contigs from patients
16S = 93.7-95.4%
16S = 98.5-99.8%
M. nonliquefaciens
Type strain
(DSM 6327T)
M. catarrhalis
Type strain
(ATCC 25238T)
16S rRNA = 98.4%
Genome ANI = 75%
16S = 99.6-100%
16S = 95.2-95.4%
Clade II
Contigs from patients
FIG S4 Diagram showing the relationships among the 2 type strains and the 2 representative contigs obtained from patient samples. The ANI value indicating genome relatedness was calculated using the complete genome sequence of M. catarrhalis RH4 strain (PRJNA 48809), which shows 100% 16S rRNA gene sequence identity with the type strain of the species.
