# Supplementary material for: Profiling bacterial community in upper respiratory tracts
Source: BMC Infect Dis. 2014 Nov 13;14:583. doi: 10.1186/s12879-014-0583-3 (PMC4236460; doi:10.1186/s12879-014-0583-3)
Supplement: Supplementary file 6 — Additional file 6: Table S2.: Comparison of major virulence genes present in M. nonliquefaciens and M. catarrhalis. The list of virulent genes was obtained from previous reports [31],[56]. The gene locus in each genome is presented together with gene size in amino acids (in parenthesis). (DOCX 22 KB) [file 12879_2014_583_MOESM6_ESM.docx]

**Table S2** Comparison of major virulence genes present in *M. nonliquefaciens* and *M. catarrhalis.* The list of virulent genes was obtained from previous reports (28, 57). The gene locus in each genome is presented together with gene size in amino acids (in parenthesis).

| Protein | General function | Locus in  *M. catarrhalis* RH4 | Locus in  *M. nonliquefaciens* DSM 6327^T^ |
| --- | --- | --- | --- |
| UspA1 | Adhesion and invasion of epithelial cells and EMC, inhibition of NF-kB proinflammatory resistance, binding of complement regulators, biofilm formation | MCR_1198 (955 aa) | MN6327_05270 (626 aa) |
| UspA2H | Adhesion to epithelial cells, serum resistance, and biofilm formation | MCR_0329 (816 aa) | - |
| MID/Hag | Adhesion to epithelial cells, IgD binding, negative effect on biofilm formation | MCR_0617 (2090 aa) | MN6327_17370 (2576 aa) |
| McaP | Adhesion to epithelial cells, lipolytic activity | MCR_0419 (650 aa) | MN6327_11560 (608 aa) |
| OmpCD | Adhesion to epithelial cells and middle ear mucin, serum resistance | MCR_1698 (453 aa) | MN6327_14130 (423 aa) |
| CopB | In vivo survival, serum resistance | MCR_0492 (759 aa) | MN6327_19250 (766 aa) |
| OmpE | Serum resistance | MCR_0858 (459 aa) | MN6327_09550 (458 aa) |
| BRO-2 | β-lactamase family protein BRO-2 | MCR_1591 (313 aa) | - |
| AmpC | β-lactamase Class C | MCR_0463 (419 aa) | MN6327_16920 (415 aa) |
| MBL | Metallo-β-lactamase family protein | - | MN6327_21920 (253 aa) |
