# Supplementary figures and images for: Profiling bacterial community in upper respiratory tracts
Source: BMC Infect Dis. 2014 Nov 13;14:583. doi: 10.1186/s12879-014-0583-3 (PMC4236460; doi:10.1186/s12879-014-0583-3)

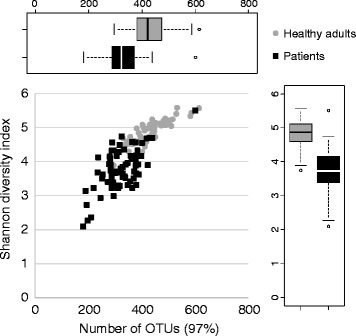

Supplement: Supplementary file 7 — Authors’ original file for figure 1 [file 12879_2014_583_MOESM7_ESM.gif]

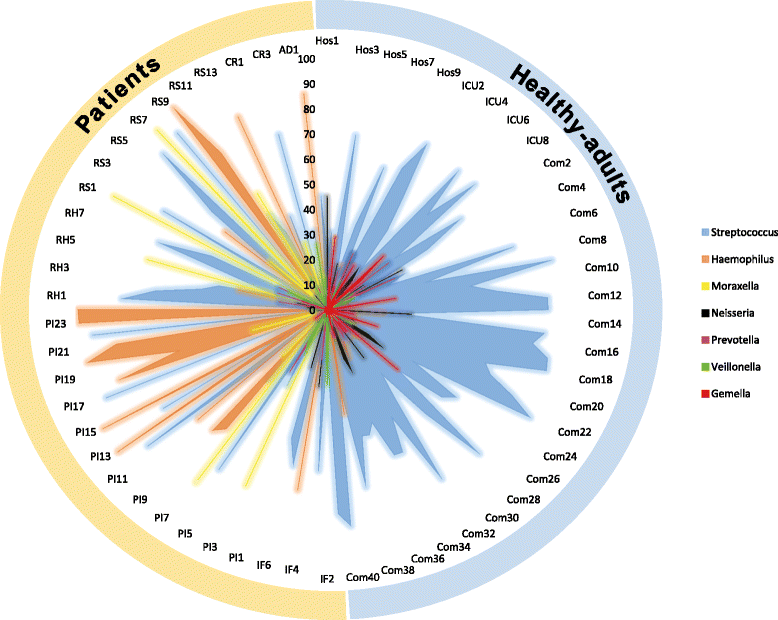

Supplement: Supplementary file 8 — Authors’ original file for figure 2 [file 12879_2014_583_MOESM8_ESM.gif]

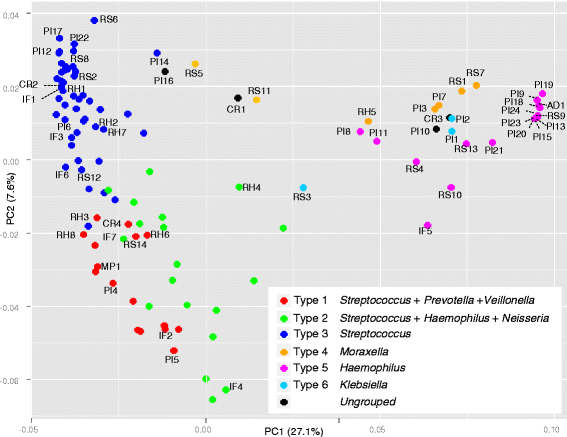

Supplement: Supplementary file 9 — Authors’ original file for figure 3 [file 12879_2014_583_MOESM9_ESM.gif]

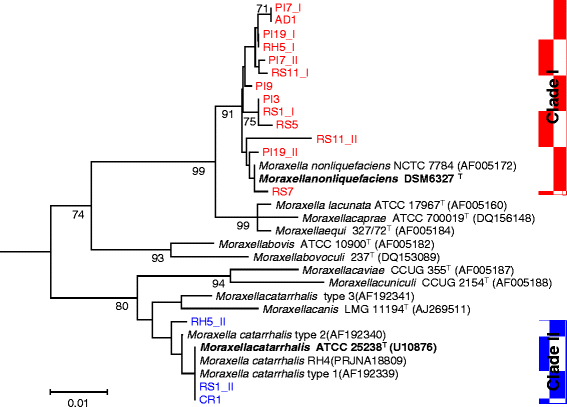

Supplement: Supplementary file 10 — Authors’ original file for figure 4 [file 12879_2014_583_MOESM10_ESM.gif]
